# Unattainable regions in the space of genetic relationships

**DOI:** 10.1007/s00285-026-02452-9

**Published:** 2026-08-06

**Authors:** Petter Mostad, Thore Egeland, Magnus Dehli Vigeland

**Affiliations:** 1 Department of Mathematical Sciences, Chalmers University of Technology and University of Gothenburg, 41296 Göteborg, Sweden; 2 Forensic Sciences, Oslo University Hospital, 0424 Oslo, Norway; 3 KBM, Norwegian University of Life Sciences, 1433 Ås, Norway

**Keywords:** Genetic relationships, IBD inequalities, Identical by state, Jacquard coefficients, Primary 92D10, Secondary 60C05

## Abstract

The relationship between two pedigree members may be summarized by their nine *condensed identity coefficients* $(\Delta _1,\dotsc ,\Delta _9)$, also known as *Jacquard coefficients*. These give the expected relative frequencies of the possible patterns of *identity by descent* between the alleles carried by the individuals at an autosomal locus. The collection *J* of all such vectors, taken over all pairs in all pedigrees, forms a subset of the probability simplex $S^8 \subset {\mathbb R}^9$. Despite decades of constant use and recurring interest in the Jacquard coefficients, remarkably little is known about the geometry of *J*. For instance, a long-standing open question is whether *J* is dense in $S^8$, or, conversely, whether there exists full-dimensional *unattainable regions* not realizable by any pedigree. In the case of non-inbred individuals, this was famously resolved by Thompson, who identified the unattainable region bounded by a quadratic equation in the two-dimensional simplex of such relationships. In this paper we develop a general framework for constructing restrictions in the identity coefficients of any number of individuals. Applied to the pairwise case, we find several explicit quadratic inequalities in the nine Jacquard coefficients, each defining a full-dimensional unattainable region. One of these contains the known region unattainable by non-inbred relationships. To our knowledge, our results provide the first examples of full-dimensional holes in the space of pairwise relationships, arising purely from Mendelian constraints.

## Introduction

The study of relationships between diploid organisms is a cornerstone of statistical genetics, with applications in a variety of fields (Jacquard 1974; Thompson 2000; Lange 2002; Butler 2014; Laporte et al. 2017). A foundational topic in this area is the inheritance at a single locus in a pedigree relating *n* individuals, in which case the relationship is described by the joint distribution of their 2*n* alleles. Alleles inherited from the same founder allele are *identical by descent* (IBD), and each realization of the inheritance process partitions the alleles into IBD classes. Such a partition is called a *detailed IBD state*.

**Fig. 1:**
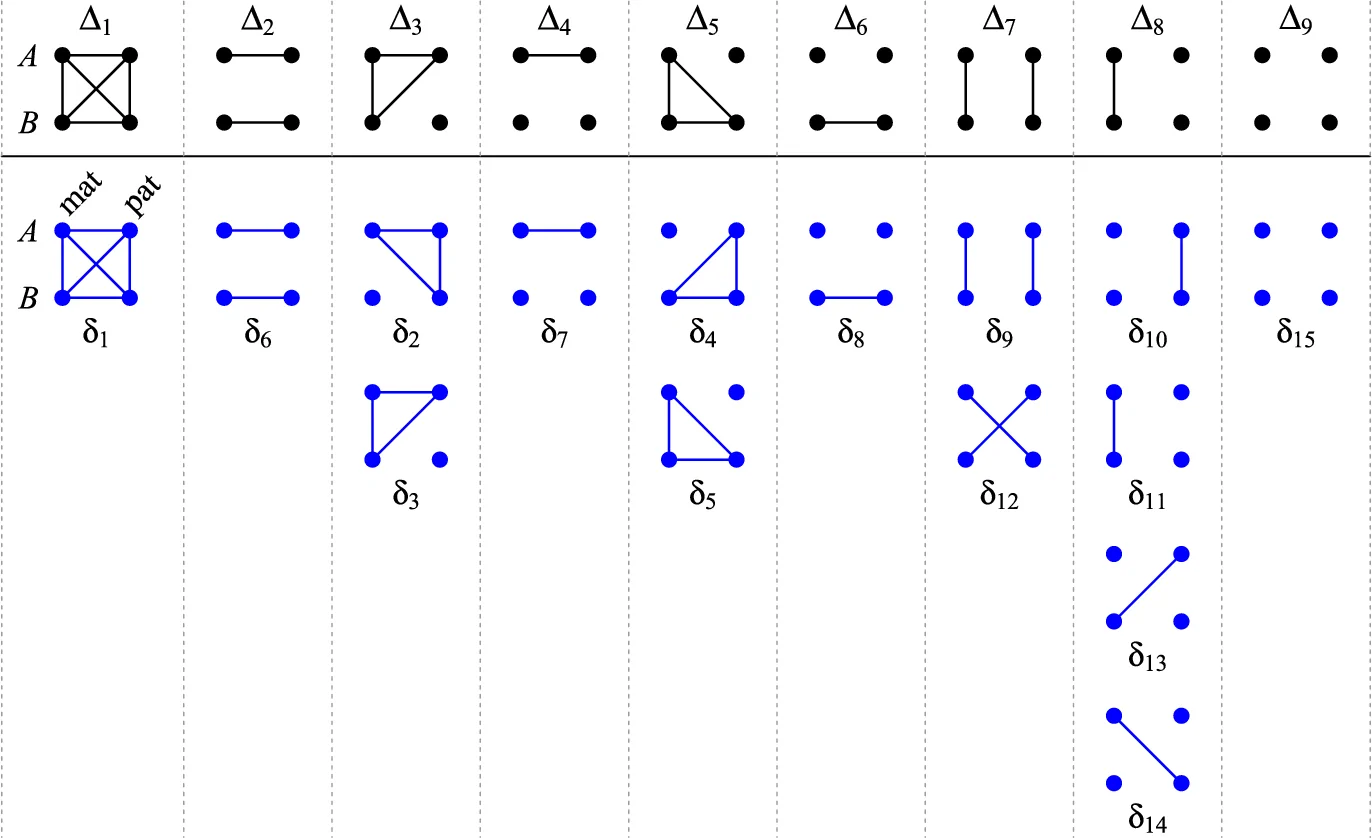
The condensed and detailed identity coefficients for two persons *A* and *B*, illustrated by the associated IBD states. The states are drawn as diagrams with four dots representing the alleles of *A* and *B*; connected dots are IBD. Each condensed state (above the horizontal line) corresponds to one or more detailed states (below the line) where the maternal/paternal alleles are distinguished. Note that we list the maternal allele before the paternal, so that the detailed figures are mirror images of those used e.g. by Jacquard (1974)

When n=2 there are 15 detailed IBD states, depicted in Fig. 1. Following Jacquard (1974), we write their probabilities as $\delta _1,\dots ,\delta _{15}$ and refer to them as *detailed identity coefficients*. For larger *n* the number of detailed states increases rapidly; see Thompson (1974) for details.

For many purposes, maternal and paternal origin of alleles is irrelevant, and it suffices to consider the two alleles of each person as an unordered pair. Detailed IBD states that differ only by swapping the two alleles within one or both individuals can then be identified, forming the *condensed IBD states* (Jacquard 1974). For two individuals there are nine such states (Fig. 1), whose probabilities $\Delta _1,\dots ,\Delta _9$ are known as the *condensed identity coefficients* or *Jacquard coefficients*. These form a point in the 8-dimensional simplex $S^8 \subset \mathbb {R}^9$.

It is natural to ask which points of $S^8$ are actually realizable as the coefficients of a pairwise relationship. While this is generally an open question, it has been completely solved for non-inbred relationships, where $\Delta _1=\dots =\Delta _6=0$. Thompson (1976) showed that all such relationships satisfy the quadratic condition

1 $$\begin{aligned} \Delta _8^2 \ge 4 \Delta _7 \Delta _9, \end{aligned}$$

which excludes two thirds of the 2-dimensional simplex as unattainable. Conversely, it can be shown that every point satisfying (1) can be realised by a non-inbred relationship, provided that infinite pedigrees are allowed  (Karigl 1984; Vigeland 2020).

Thompson and others subsequently pursued the unattainability problem in a series of papers (Thompson 1978, 1980, 1981; Karigl 1984). The most extensive of these (Thompson 1980) gives a detailed analysis of the case where one individual is a direct (non-collateral) descendant of the other. By re-parametrizing this subset of $S^8$ in terms of five interpretable parameters, several constraints are derived, prompting a conclusion that within this 5-dimensional space, the actually attainable set is only a small subset which “is neither of simple form nor can it be readily re-parametrized to become so”. Together, these works make it clear that Mendelian segregation imposes strong geometric restrictions on the set *J* of condensed identity coefficients. Still, no unconditional constraints for the full set $J \subseteq S^8$ seem to be known in the literature. Indeed, the following quote (Thompson 1986) appears valid: “Another problem is that the attainable space of gene identities probabilities, not yet fully known for two individuals, remains, for more than two, a completely unknown animal”.

In this paper we develop a framework for constructing restrictions on the detailed and condensed identity coefficients for any number of individuals. Applied to the two-person case, we obtain several explicit quadratic inequalities in the nine coefficients $\Delta _1,\dotsc , \Delta _9$, each inequality defining a full-dimensional unattainable region in $S^8$.

Section 2 reviews the literature on generalized kinship coefficients, providing background and context for the concepts and notation introduced in Sect. 3. Sect. 4 then focuses on the pairwise case (n=2), preparing examples and applications in later sections. Our main theorem is presented in Sect. 5, and applied in the pairwise case in Sect. 6. Certain proofs and details are deferred to Sect. 7. We end with some concluding remarks in Sect. 8.

## Overview of generalized kinship coefficients

Over the years, numerous algorithms have been proposed for computing the detailed (δ) and condensed (Δ) identity coefficients. Most of these methods rely on a broader class of quantities known as *generalized kinship coefficients* (GKCs), which play a substantial role also in the present work. In this section we give a brief overview of the different types of GKCs and how they appeared in the literature.

Recall that the “classical” kinship coefficient $\varphi _{ab}$ between two pedigree members *a* and *b* is the probability that a random allele from *a* is IBD with a random allele from *b* sampled at the same (autosomal) locus. In the notation to be introduced shortly, this is written $\varphi _{ab} = \Phi ([a,b])$. Note that the idea behind the kinship coefficient extends naturally to more than two individuals. For example, we denote by Φ([a,b,c]) the probability that homologous alleles drawn from *a*, *b* and *c* are all IBD. More than one IBD group can also be included: Φ([a,b],[c,d]) is the probability that alleles from *a* and *b* are IBD and alleles from *c* and *d* are IBD. In this case we must also decide whether or not IBD *between* the groups should be allowed; this choice gives rise to different types of coefficients (see below).

A *generalized IBD pattern* associated with a pedigree, consists of a collection of subsets of homologous alleles,

2 $$\begin{aligned} \mathcal {G}= \{[a_1, \dotsc , a_n], [a_{n+1}, \dotsc , ], \dotsc [ \dotsc , a_N ]\} \end{aligned}$$

where the alleles indicated by each subset, or *block*, are IBD. In this notation, following Vigeland (2023), each symbol $a_i$ is the name of a pedigree member, but within a generalized IBD pattern it refers to an allele of that individual. An individual $a_i$ may appear in multiple blocks, and also more than once within a block. The associated *generalized kinship coefficient* $\Phi (\mathcal {G})$ is the probability of observing the pattern $\mathcal {G}$ in the pedigree.

Importantly, generalized IBD patterns and their coefficients come in different flavors. We distinguish between *distinct* patterns, where alleles in different blocks are not IBD, and *non-distinct* patterns, where alleles in different blocks may be IBD. In addition, patterns can be *random*, where each allele is randomly sampled from its carrier, or *deterministic*, where the parental origin is specified. In the latter case, the extra information is added with superscripts *m* (maternal) and *p* (paternal); for example, $\mathcal {G}= \{[a^m, a^p]\}$ means that *a* is autozygous (so that $\Phi (\mathcal {G})$ equals the inbreeding coefficient $f_a$). If the parental origin is specified only for some alleles, while others are random, the pattern is *partially*, rather than *fully*, deterministic.

The notion of generalized kinship coefficients originated with Karigl (1981), who used a selection of (what we call) non-distinct, random GKCs to develop a recursive algorithm for the condensed identity coefficients $\Delta _1, \dotsc , \Delta _9$. Weeks and Lange (1988) extended this to distinct, random GKCs, and gave a general algorithm for computing these. In a follow-up paper, Lange and Sinsheimer (1992) clarified the difference between distinct and non-distinct GKCs and showed that distinctness simplifies and improves the algorithm of Karigl (1981). In the same paper they also introduced deterministic, distinct GKCs, and used them to compute the detailed identity coefficients $\delta _1, \dotsc , \delta _{15}$. Weeks et al. (1995) later extended these methods to X-chromosomal loci. Completing the picture, García-Cortés (2015) considered non-distinct, fully deterministic GKCs and used these to obtain an alternative algorithm for the detailed identity coefficients.

Extensions of GKCs to more than one locus have also been studied. For details about this we refer to Vigeland (Vigeland 2023) and references therein.

Many examples of GKCs are given in the documentation of the R package ribd (Vigeland 2021). Moreover, the function gKinship() of this package implements all the flavors of GCKs described above.

In the present work, the relevant class is that of ‘distinct, deterministic’ GKCs. Crucially, the detailed IBD coefficients between *n* pedigree members are special cases of such GKCs, whose IBD pattern (2) contains precisely the 2*n* alleles of the individuals. In the next section, we introduce a compact notation that is particularly well suited to this setting.

## The indexing notation

Consider an ordered list of the *n* allele pairs of *n* persons in a pedigree. We use the convention that for each allele pair, the maternal allele is always listed first. Building on Mostad et al. (2023), we use a string of 2*n* symbols to describe a set of disjoint subsets of positions in this list. Each symbol is either a non-negative integer or the letter *x*. In the text below, no integers above 9 occur, so we can write the list as a string of back-to-back symbols without creating ambiguity. Each integer value specifies a subset of the alleles. Thus, unless the list contains an *x*, it specifies a detailed IBD state.

For example, the string 0101 describes the detailed IBD state for two persons at a locus where their maternal alleles are IBD, and their paternal alleles are also IBD (but not IBD with the maternal alleles). In the notation of the previous section this corresponds to the pattern $\{[a^m, b^m], [a^p, b^p]\}$, assuming the persons are named *a* and *b*. When we want to describe a partition of only a subset of the 2*n* alleles, we use the letter *x* to fill in the remaining positions. For example, 00*xx* describes the detailed IBD state for the first person where this person is autozygous. (In the previous section this would be written $\{[a^m, a^p]\}$.). Note that *x* simply indicates "no information", so that 00*xx* corresponds to the set of detailed IBD states 0000, 0001, 0010, 0011, and 0012 for the two persons.

According to the definitions above, the same set of subsets of allele positions could be represented by many different strings: Instead of 0101 we could also use 1010, 3232, or 5959. From now on, we choose to always use the string that comes first in the natural sort order of the possible strings, i.e., when we first sort according to the first symbol, then according to the second symbol, etc. Such a string will be called an *indexing*. Thus 0101 is an indexing, while 1010 and 3232 are not. Similarly, 100*x* is not an indexing, while its equivalent string 011*x* is.

Given a pedigree and an indexing *I*, we denote by Φ[I] the corresponding GKC, that is, the probability that alleles inherited according to the pedigree will be IBD according to the subsets given by *I*. We extend the notation to sets of indexings, so that we write for example

3 $$\begin{aligned} \Phi [011x] = \Phi [0110, 0111, 0112] = \Phi [0110] + \Phi [0111] + \Phi [0112]. \end{aligned}$$

Note that the first equality will be seen to be a consequence of Lemma 1 while the second indicates how we apply Φ to sets of indexings.

As mentioned in Sect. 1, a condensed IBD state is an equivalence class of detailed IBD states that can be obtained from each other using switches of paternal and maternal alleles. So, for example, the detailed IBD states 0102, 0112, 0120, and 0121 constitute a single condensed IBD state, which we denote using curly brackets around the earliest indexing in the natural sort order, i.e., writing $\{0102\}$ in this case. For probabilities we still use Φ, so that, for example,

4 $$\begin{aligned} \Phi \{0102\} = \Phi [0102] + \Phi [0112] + \Phi [0120] + \Phi [0121]. \end{aligned}$$

## Pairwise identity coefficients

Figure 1 illustrates the detailed identity coefficients $\delta _1,\dots ,\delta _{15}$ and the condensed identity coefficients $\Delta _1,\dots ,\Delta _9$ for two individuals, in the ordering of Jacquard (1974). The connection between the traditional notation and our Φ-notation is

5 $$\begin{aligned} \begin{aligned} \Phi \{0000\} = \Delta _1&= \delta _1 = \Phi [0000]\\ \Phi \{0011\} = \Delta _2&= \delta _6 = \Phi [0011] \\ \Phi \{0001\} = \Delta _3&= \delta _2 + \delta _3 = \Phi [0010] + \Phi [0001] \\ \Phi \{0012\} = \Delta _4&= \delta _7 = \Phi [0012] \\ \Phi \{0100\} = \Delta _5&= \delta _4 + \delta _5 = \Phi [0111] + \Phi [0100] \\ \Phi \{0122\} = \Delta _6&= \delta _8 = \Phi [0122] \\ \Phi \{0101\} = \Delta _7&= \delta _9 + \delta _{12} = \Phi [0101] + \Phi [0110] \\ \Phi \{0102\} = \Delta _8&= \delta _{10} + \delta _{11} + \delta _{13} + \delta _{14} \\&= \Phi [0121] + \Phi [0102] + \Phi [0112] + \Phi [0120] \\ \Phi \{0123\} = \Delta _9&= \delta _{15} = \Phi [0123], \end{aligned} \end{aligned}$$

where, in each line, the $\Phi [\cdot ]$-terms follow the same order as the corresponding $\delta _i$-terms.

Several other standard quantities concerning pairwise relationships can also be expressed in our Φ-notation. We collect some of these here, including their relations to the identity coefficients. First, the inbreeding coefficient of each person is

6 $$\begin{aligned} \begin{aligned} \Phi [00xx]&= f_1 = \Delta _1 + \Delta _2 + \Delta _3 + \Delta _4 \\ \Phi [xx00]&= f_2 = \Delta _1 + \Delta _2 + \Delta _5 + \Delta _6. \end{aligned} \end{aligned}$$

The right-hand sides follow from Fig. 1 by summing the condensed coefficient for which the respective individual is autozygous. We also record the complementary probabilities,

7 $$\begin{aligned} \begin{aligned} \Phi [01xx]&= \bar{f_1} = 1 - f_1 = \Delta _5 + \Delta _6 + \Delta _7 + \Delta _8 + \Delta _9\\ \Phi [xx01]&= \bar{f_2} = 1 - f_2 = \Delta _3 + \Delta _4 + \Delta _7 + \Delta _8 + \Delta _9. \end{aligned} \end{aligned}$$

Finally, we have the following coefficients for IBD between specific alleles:

8 $$\begin{aligned} \begin{aligned} \Phi [0x0x]&= q_{MM} = \delta _1 + \delta _3 + \delta _5 + \delta _9 + \delta _{11} \\ \Phi [0xx0]&= q_{MP} = \delta _1 + \delta _2 + \delta _5 + \delta _{12} + \delta _{14} \\ \Phi [x00x]&= q_{PM} = \delta _1 + \delta _3 + \delta _4 + \delta _{12} + \delta _{13} \\ \Phi [x0x0]&= q_{PP} = \delta _1 + \delta _2 + \delta _4 + \delta _9 + \delta _{10}. \end{aligned} \end{aligned}$$

When neither of the two individuals are ancestors of each other, the coefficients in (8) are the kinship coefficients between the parents: $q_{MM}$ is the kinship coefficient between the mothers, and similarly for the other pairs. The sums on the right-hand side again follow from Fig. 1; for instance, $q_{MM}$ corresponds to the detailed states in which the two maternal alleles are connected.

We can use the Eq. (8) to recover a well-known identity expressing the kinship coefficient φ between two individuals in terms of their condensed identity coefficients. Observe first that

9 $$\begin{aligned} 4\varphi = q_{MM}+q_{MP}+q_{PM}+q_{PP}, \end{aligned}$$

since the four terms correspond to the possible choices of one allele from each individual. Therefore, summing the equations in (8) gives

10 $$\begin{aligned} \begin{aligned} 4\varphi&= 4\delta _1 + 2\delta _2 + 2\delta _3 + 2\delta _4 + 2\delta _5 + 2\delta _9 + \delta _{10} + \delta _{11} + 2\delta _{12} + \delta _{13} + \delta _{14} \\&= 4\Delta _1 + 2\Delta _3 + 2\Delta _5 + 2\Delta _7 + \Delta _8. \end{aligned} \end{aligned}$$

For more details about this and other formulas involving identity coefficients, we refer to Jacquard (1974).

### Restrictions

Let *J* denote the set of all points $(\Delta _1, \dotsc , \Delta _9) \in \mathbb {R}^9$ that are the condensed identity coefficients of some pairwise relationship. To avoid complications in the ensuing discussion we allow infinite pedigrees in this definition, so that *J* contains all of its limit points. By definition, *J* is contained in the probability simplex $S^8\subseteq \mathbb {R}^9$. Our overall aim is to understand the geometry of $J \subseteq S^8$.

In the following, a *restriction* $\mathcal {R}$ refers to a finite collection of inequalities $F_i(\Delta _1,\dots ,\Delta _9)\ge 0$ that hold for all points of *J*, possibly conditional on a finite set of equalities $G_j(\Delta _1,\dots ,\Delta _9)=0$. For example, Thompson’s restriction for noninbred individuals is given by the single inequality $\Delta _8^2 - 4\Delta _7\Delta _9 \ge 0$, conditional on the six equalities $\Delta _1 = \cdots = \Delta _6 = 0$. In the present work, the functions $F_i$ are polynomials, while the constraints $G_j = 0$ correspond to facets of $S^8$, but this is not essential.

For any restriction $\mathcal {R}$ we associate its *unattainable region* $T_\mathcal {R}\subseteq \mathbb {R}^9$, defined by the reversed inequalities $F_i<0$ together with the (unchanged) equalities $G_j=0$. We call $\mathcal {R}$ *non-trivial* if it excludes at least one point of the simplex, that is, $T_\mathcal {R}\cap S^8 \ne \varnothing$. Furthermore, $\mathcal {R}$ is *global* if its unattainable region is full-dimensional, $\dim (T_\mathcal {R}) = 9$. In particular, a global restriction has no equality conditionals. It should also be noted that if a global $\mathcal {R}$ is non-trivial, its unattainable region $T_\mathcal {R}$ in fact contains an open subset of $S^8$. (This assumes that $\mathcal {R}$ is defined by non-strict inequalities $F_i \ge 0$, which will be the case in all of our examples.)

A restriction $\mathcal {R}_1$ *contains* another restriction $\mathcal {R}_2$, written $\mathcal {R}_1 \succeq \mathcal {R}_2$, if $T_{\mathcal {R}_1} \supseteq T_{\mathcal {R}_2}$. If neither $\mathcal {R}_1 \succeq \mathcal {R}_2$ nor $\mathcal {R}_2 \succeq \mathcal {R}_1$, the two restrictions are *non-redundant*.

## The product theorem

We now introduce products of indexings, i.e. ways of combining two indexings. These are the ingredients in our first main result, the Product Theorem, stated at the end of the section.

The *support* of an indexing *I* is the set of positions not containing *x*’s. The size of the support is denoted $$\operatorname {Supp}\left( I \right) $$. For indexings *I* and $I^{\prime }$ with disjoint supports, we consider two different product operations. First, we define the *dot product* $I\cdot I^{\prime }$ as the indexing that simply joins the two sets of subsets indicated by *I* and $I^{\prime }$. More formally, one can obtain $I\cdot I^{\prime }$ by first changing the integers of $I^{\prime }$ to values larger than those of *I*, and inserting them in their corresponding positions of *I* (which were *x*’s before, since *I* and $I^{\prime }$ have disjoint support). Finally, the resulting string is converted to its indexing representative. The resulting dot product is clearly commutative, i.e., $$I \cdot I' = I'\cdot I$$. As some small examples, we have

$$\begin{aligned} 0xx0\cdot x00x= &  0110 \\ xx01\cdot 0xxx= &  0x12 \\ 0xx1\cdot x01x= &  0123. \end{aligned}$$

Informally, the dot product $I\cdot I^{\prime }$ can be thought of as a simple concatenating operator, collecting the allelic identities prescribed by *I* *and* those prescribed by $I^{\prime }$, with no mixing between the two.

Next, we define a *wedge product* $I\wedge I^{\prime }$ of indexings *I* and $I^{\prime }$ with disjoint supports. Unlike the dot product, the wedge product $I\wedge I^{\prime }$ is generally a set of multiple indexings, representing all the different ways in which $I^{\prime }$ can be merged into and combined with *I*. It is derived as follows: First, all integers in $I^{\prime }$ are replaced with integers higher than those in *I*. Then, for each of the positions in the support of $I^{\prime }$, the value is either kept or replaced with one of the integers of *I*. The two indexings are then merged in a single string, which is finally replaced with its indexing representative. Two examples are

11 $$\begin{aligned} \begin{aligned} 00xx \wedge xx00&= \{0000, 0001, 0010, 0011\} \\ xx00 \wedge 00xx&= \{0000, 0100, 0111, 0011\}. \end{aligned} \end{aligned}$$

As a worked example, 001xxx∧xxx011 is computed by first replacing *xxx*011 with *xxx*233. Then, replacing each of the three integers in the string as described, we get

$$\begin{aligned}&\{ 001000, 001001, 001003, 001010, 001011, 001013, 001030, 001031, 001033,&\\&001100, 001101, 001103, 001110, 001111, 001113, 001130, 001131, 001133,&\\&001200, 001201, 001203, 001210, 001211, 001213, 001230, 001231, 001233\}.&\end{aligned}$$

Finally, replacing each of these strings with their indexing representative yields the result

12 $$\begin{aligned} \begin{aligned} 001xxx\wedge xxx011 = \{&001000, 001001, 001002, 001010, 001011, 001012,\\&001020, 001021, 001022, 001100, 001101, 001102,\\&001110, 001111, 001112, 001120, 001121, 001122,\\&001200, 001201, 001203, 001210, 001211, 001213,\\&001230, 001231, 001233 \}. \end{aligned} \end{aligned}$$

Note that the wedge product does not introduce any new IBD connections in the support of *I*, and in the support of $I^{\prime }$ such connections are introduced only for positions if they also become IBD with positions in *I*. Note also that the number of indexings in $I\wedge I^{\prime }$ is $$(i+1)^{\operatorname {Supp}\left( I' \right) }$$, where *i* is the number of different integers in *I*. For example, the last wedge result above had $$(2+1)^3 = 27$$ members. Finally, note that $$I\cdot I'\in I\wedge I'$$. It is important to remember that the wedge product is *not* commutative, so that in general $I\wedge I^{\prime }$ is not equal to $I^{\prime }\wedge I$.

When $$\operatorname {Supp}\left( I' \right) =1$$, the wedge product simply lists the ways in which the single position in the support of $I^{\prime }$ can either be IBD with the IBD groups in *I* or not IBD with any of them. Thus the law of total probability yields the following Lemma:

### Lemma 1

Given any pedigree and indexings *I* and $I^{\prime }$ involving the alleles of *n* of its members. Assume the supports are disjoint and that $$\operatorname {Supp}\left( I' \right) =1$$. We then have

$$ \Phi [I] = \Phi [I\wedge I']. $$

Basic results like Eq. (3) can be formally obtained using the Lemma with I=011x and $I^{\prime }=xxx0$. Similarly one may prove identities like (6), (7), (8) by applying the Lemma twice.

We are now ready to state our main theorem, whose proof can be found in Sect. 7.

### Theorem 1

Given any pedigree and indexings *I* and $I^{\prime }$ relating *n* persons. If the supports are disjoint, we have

13 $$\begin{aligned} \Phi [I\cdot I'] \le \Phi [I]\Phi [I']\le \Phi [I\wedge I']. \end{aligned}$$

In fact, by switching *I* and $I^{\prime }$, we immediately obtain the following stronger formulation:

14 $$\begin{aligned} \Phi [I\cdot I'] \le \Phi [I]\Phi [I']\le \min \left( \Phi [I\wedge I'], \Phi [I'\wedge I]\right) . \end{aligned}$$

## Applications to pairwise relationships

In this section, we use Theorem 1 to derive several global restrictions in the pairwise coefficients $\Delta _1,\dots ,\Delta _9$. For compactness, we occasionally use the shorthand notation $\Delta _{1+2} := \Delta _1+\Delta _2$, and similarly for other sums.

### Theorem 2

For any pairwise relationship the following inequalities hold:

15 $$\begin{aligned} -\min (\Delta _3, \Delta _5) \;\le \; \Delta _{1+2}\Delta _{7+8+9} - \Delta _{3+4}\Delta _{5+6} \;\le \;\min (\Delta _1, \Delta _{7+8}) \end{aligned}$$

Moreover, each inequality is a non-trivial global restriction, and neither is contained in the other.

### Proof

To start off, consider the indexings I=00xx and $I^{\prime }=xx00$. Then $I\cdot I^{\prime }=0011$, while the wedge products $I\wedge I^{\prime }$ and $I^{\prime }\wedge I$ are given in (11). Hence, by the product theorem,

$$\begin{aligned}\begin{aligned} \Phi [0011]&\le \Phi [00xx]\Phi [xx00] \\&\le \Phi [0000] + \Phi [0011] + \min (\Phi [0001]+\Phi [0010], \Phi [0100]+\Phi [0111]), \end{aligned} \end{aligned}$$

which translates into

16 $$\begin{aligned} \Delta _2 \le f_1f_2 \le \Delta _1 + \Delta _2 + \min (\Delta _3, \Delta _5). \end{aligned}$$

Since, by (6), each $$f_i$$ may be expressed in $$\Delta _i$$’s, each side of (16) gives an inequality purely in $$\Delta _i$$’s. Similarly, with I=01xx and $I^{\prime }=xx01$, and the expressions for $$\bar{f_i} = 1-f_i$$ in (7), we find:

17 $$\begin{aligned} \Delta _9 \le \Phi [01xx]\Phi [xx01] = \bar{f_1}\bar{f_2} \le \Delta _7+\Delta _8+\Delta _9+\min (\Delta _3,\Delta _5). \end{aligned}$$

Finally, we can pair up the indexings used above in two more ways, producing the following:

18 $$\begin{aligned} \Delta _4&\;\le \; \Phi [00xx]\Phi [xx01] = f_1 \bar{f_2} \;\le \; \Delta _3+\Delta _4 + \min (\Delta _1, \Delta _7 + \Delta _8) \end{aligned}$$

19 $$\begin{aligned} \Delta _6&\;\le \; \Phi [01xx]\Phi [xx00] = \bar{f_1} f_2 \;\le \; \Delta _5+\Delta _6 + \min (\Delta _1, \Delta _7 + \Delta _8). \end{aligned}$$

Altogether, (16), (17), (18), (19) provide 8 inequalities in the Δ’s, all of which can be shown to give non-trivial global restrictions. However, several of these are redundant. Let (16a) and (16b) denote, respectively, the left and right inequalities in (16), and similarly for (17), (18), (19). Then it is straightforward to prove the following sets of implications:

20 $$\begin{aligned} \begin{aligned}&(16b) \;\Longleftrightarrow \; (17b) \; \Longrightarrow \; (18a),\; (19a) \\&(18b) \;\Longleftrightarrow \; (19b) \; \Longrightarrow \; (16a),\; (17a). \end{aligned} \end{aligned}$$

For example, to show that (16b)⟹(18a) it is enough to observe that if (16b) holds, then

$$\begin{aligned} \begin{aligned} f_1\bar{f_2}&= f_1(1-f_2) = f_1 - f_1f_2 \\&\ge (\Delta _1 + \Delta _2 +\Delta _3 +\Delta _4) - (\Delta _1 + \Delta _2 + \min (\Delta _3, \Delta _5)) \\&= \Delta _3 + \Delta _4 - \min (\Delta _3, \Delta _5) \\&\ge \Delta _4, \end{aligned} \end{aligned}$$

where the assumption (16b) was used in the first inequality. The other implications are proved similarly.

It follows from (20) that all 8 restrictions are contained in those given by (16b) and (18b), namely (using the shorthand notation)

$$\begin{aligned} \Delta _{1+2+3+4}\Delta _{1+2+5+6}&\le \Delta _1 + \Delta _2 + \min (\Delta _3, \Delta _5) \\ \Delta _{1+2+3+4}\Delta _{3+4+7+8+9}&\le \Delta _3 + \Delta _4 + \min (\Delta _1, \Delta _7 + \Delta _8). \end{aligned}$$

After some rearranging (and using $\sum \Delta _i = 1$), these can be written as, respectively,

21 $$\begin{aligned} \Delta _{3+4}\Delta _{5+6}&\;\le \; \Delta _{1+2}\Delta _{7+8+9} + \min (\Delta _3, \Delta _5) \end{aligned}$$

22 $$\begin{aligned} \Delta _{1+2}\Delta _{7+8+9}&\;\le \; \Delta _{3+4}\Delta _{5+6} + \min (\Delta _1, \Delta _{7+8}) , \end{aligned}$$

which is immediately equivalent with (15).

Finally, we must show that (21) and (22) give non-trivial and non-redundant restrictions. To this end, consider the following points, which are both probability vectors in $S^8 \subseteq \mathbb {R}^9$:

$$\begin{aligned} P_1&= (0.02, 0.11, 0.19, 0.21, 0.09, 0.20, 0.09, 0.08, 0.01) \\ P_2&= (0.04, 0.20, 0.03, 0.06, 0.08, 0.20, 0.03, 0.14, 0.22) \end{aligned}$$

One can check that $P_1$ violates (21) but not (22), and *vice versa* for $P_2$. This concludes the proof. □

As evident from the above proof, the restrictions in Theorem 2 are connected with the inbreeding coefficients of the individuals. We now prove another restriction, related to their kinship coefficient φ.

### Theorem 3

For any pairwise relationship the following inequality holds:

23 $$\begin{aligned} (4\Delta _1 + 2\Delta _3 + 2\Delta _5 + 2\Delta _7 + \Delta _8)^2 \ge 4\Delta _7, \end{aligned}$$

or, equivalently,

24 $$\begin{aligned} 4\varphi ^2 \ge \Delta _7. \end{aligned}$$

This gives a non-trivial global restriction, independent of those in Theorem 2.

### Proof

To demonstrate (24), we start by squaring the identity $4\varphi = q_{MM} + q_{PP} + q_{MP} + q_{PM}$ from (9), and applying standard algebraic inequalities:

25 $$\begin{aligned} \begin{aligned} (4\varphi )^2&= (q_{MM} + q_{PP} + q_{MP} + q_{PM})^2 \\&\ge (q_{MM} + q_{PP})^2 + (q_{MP} + q_{PM})^2 \\&\ge 4q_{MM}q_{PP} + 4q_{MP}q_{PM}. \end{aligned} \end{aligned}$$

Each of these final terms can be controlled by invoking the left side of the Product theorem:

26 $$\begin{aligned} \begin{aligned} q_{MM}q_{PP}&= \Phi [0x0x]\Phi [x0x0] \,\ge \, \Phi [0101] = \delta _9 \\ q_{MP}q_{PM}&= \Phi [0xx0]\Phi [x00x] \,\ge \, \Phi [0110] = \delta _{12}. \end{aligned} \end{aligned}$$

Combining these with (25) we get

27 $$\begin{aligned} (4\varphi )^2 \ge 4q_{MM}q_{PP} + 4q_{MP}q_{PM} \ge 4\delta _9 + 4\delta _{12} = 4\Delta _7, \end{aligned}$$

from which (24) follows. The equivalence of Eqs. (23) and (24) follows from Eq. (10).

To prove non-triviality, one can easily verify that (23) is violated by the probability vector

$$\begin{aligned} P_3 = (0.06, 0.12, 0.06, 0.23, 0.03, 0.21, 0.20, 0.04, 0.05). \end{aligned}$$

Furthermore, $P_3$ does not violate any of the inequalities of Theorem 2, nor do either of $P_1$ and $P_2$ violate (23), which shows that the two Theorems give non-redundant restrictions. □

Note that the restriction given by Theorem 3 contains Thompson’s restriction for non-inbred individuals. Indeed, setting $\Delta _1 = \cdots = \Delta _6 = 0$ in Eq. (23) yields

$$\begin{aligned} 4\Delta _7^2 + 4\Delta _7\Delta _8 + \Delta _8^2\ge &  4 \Delta _7 \\ \Delta _8^2\ge &  4\Delta _7(1-\Delta _7-\Delta _8) \\ \Delta _8^2\ge &  4\Delta _7\Delta _9. \end{aligned}$$

It may be of interest to quantify the sizes of the unattainable regions we have identified, analogous to Thompson’s restriction which excludes two thirds of the noninbred relatedness triangle (Thompson 1976). Let *A* and *B* be the unattainable regions corresponding to the restrictions in Theorem 2 (*A* for the left-hand inequality and *B* for the right), and let *C* be the unattainable region established in Theorem 3. As exact calculations are difficult, we estimated their relative volumes within the simplex $S^8$ by Monte Carlo, sampling $10^6$ points from the uniform Dirichlet(1,⋯,1) distribution. The estimated volume fractions of $S^8$ were:

$$\begin{aligned} \textrm{Vol}_{S^8}(A)&\approx 0.114,\\ \textrm{Vol}_{S^8}(B)&\approx 0.182,\\ \textrm{Vol}_{S^8}(C)&\approx 0.064. \end{aligned}$$

While *A* and *B* are obviously disjoint, region *C* overlaps markedly with both, with $\Pr (A\cap C) \approx 0.012$ and $\Pr (B\cap C) \approx 0.034$.

## Theory and proofs

We will work with *allelic pedigrees*, defined as directed acyclic graphs where each node has either zero or two directed edges pointing towards it. An ordinary pedigree can be mapped to an allelic pedigree by mapping each person to two nodes, representing their maternal and paternal alleles, and mapping each parent-child relationship to the pair of edges representing how an allele is inherited either from the maternal or the paternal allele of the parent. However, we note that the set of allelic pedigrees also contains graphs that cannot be mapped from ordinary pedigrees. For example, if three nodes have parent nodes in a set of three other nodes such that each child node has a different parent pair, there is no way to assign genders so that each parent pair contains two genders.

**Fig. 2:**
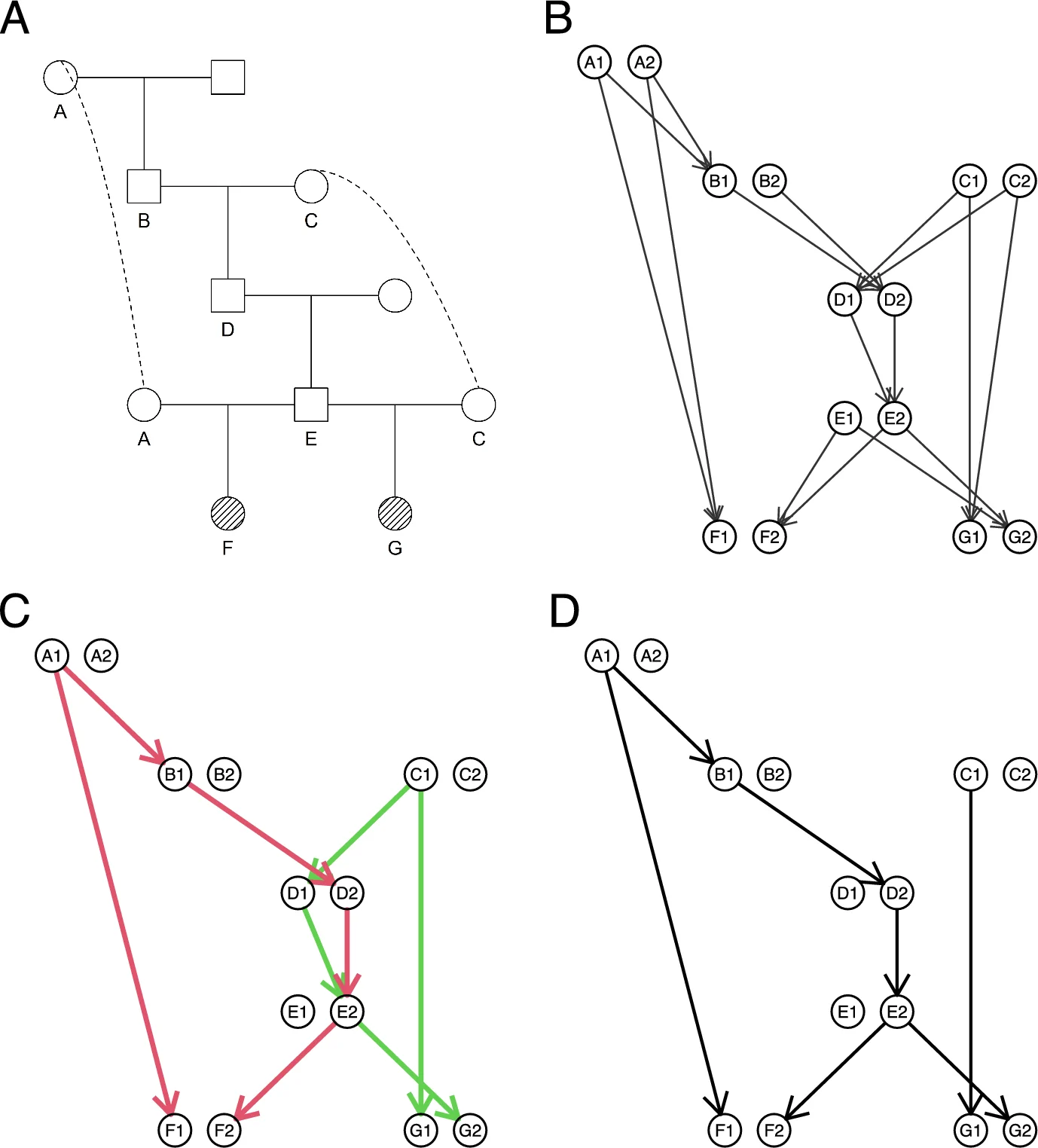
Panel A shows an ordinary pedigree, relating individuals F and G. Panel B shows the corresponding allelic pedigree. Note that we do not include the alleles of founder individuals with only one child, as these do not influence the identity coefficients. Panel C shows two IBD trees $P_1\in \mathcal{P}_{00xx}$ (red) and $P_2\in \mathcal{P}_{xx00}$ (green). Their wedge product $P_1\wedge P_2\in \mathcal{P}_{0010}$ is shown in Panel D

Figure 2 shows an ordinary pedigree in panel A, and its corresponding allelic pedigree in panel B. This somewhat complex pedigree was chosen to illustrate the concept of ‘hijacking’ introduced below. Note that the transmission from from D to E acts like a switch, passing on either allele D1 (from individual C) or allele D2 (from individual B). As a result, the children F and G cannot be autozygous simultaneously, although both are inbred. In other words, their relationship have Jacquard coefficients $\Delta _1 = \Delta _2 = 0$, while both $f_F > 0$ and $f_G > 0$. (This is the simplest relationship we know to have this property.)

An *inheritance pattern* is, for each of the *K* nodes that have two edges pointing into it, a choice of one of those edges. Biologically, it corresponds to a description of how alleles may have been inherited through the pedigree at a particular locus. The set $\mathcal{P}$ of possible inheritance patterns has size $2^K$. We assign equal probability to all inheritance patterns, so each has probability $2^{-K}$.

To any allelic pedigree we associate an ordered list of *focus* nodes. These will usually be the maternal and paternal alleles of persons whose relationship we are considering, but the theory works for any selection of nodes. We call nodes with zero incoming edges *founder* nodes, and we use the word *path* for directed paths (in the graph-theoretical sense) that start at a founder node and end at a focus node. A path can be identified with the subset $P\subseteq \mathcal{P}$ of all inheritance patterns containing the edges of the path. If the path contains *k* edges, *P* will have size $2^K/2^k = 2^{K-k}$ and probability $2^{-k}$. If we for example have 6 focus nodes, we use the notation $\mathcal{P}_{xx0xxx}$ for the set of paths that end at the third focus node, and similar notation $\mathcal{P}_{x\dots x0x\dots x}$ for other cases. Note that each such set $\mathcal{P}_{x\dots x0x\dots x}$ consists of disjoint sets whose union is $\mathcal{P}$.

Given one or more focus nodes, an *IBD tree* with these nodes as *support* consists of paths that all start at the same founder node (which may be an ancestor of the node where paths diverge) and end at each of the nodes in the support. The red line in panel C of Fig. 2 illustrates an IBD tree relating the first and second focus nodes, while the green line illustrates an IBD tree relating the third and fourth focus nodes. Note that, if an inheritance pattern contains an IBD tree, alleles inherited with this pattern will be identical by descent. As an example with 6 focus nodes, the set of IBD trees ending at the second and fourth focus nodes is denoted $\mathcal{P}_{x0x0xx}$, with similar notation in other cases. If an IBD tree has *k* edges in total, the corresponding subset $P\subseteq \mathcal{P}$ has size $2^{K-k}$ and probability $2^{-k}$. Note also that, while for example $\mathcal{P}_{0x0x\dots x}$ consists of disjoint subsets, their union is not $\mathcal{P}$ but the set of all inheritance patterns for which the first and third focus nodes are IBD.

IBD trees are called *separate* if they do not intersect at any node. In panel C of Fig. 2, the green and red IBD trees are *not* separate. Given separate IBD trees $P_1, P_2, \dots , P_s$ we define their *IBD forest* as the union of their edges, or, when considering them as subsets of $\mathcal{P}$, their intersection $P_1\cap P_2\cap \dots \cap P_s$. Panel D of Fig. 2 shows an IBD forest consisting of one IBD tree in $\mathcal{P}_{00x0}$ and a separate IBD tree in $\mathcal{P}_{xx0x}$. If *I* is an indexing as introduced in Sect. 3, we let $\mathcal{P}_{I}$ denote the set of IBD forests which for each integer in *I* has a separate IBD tree connecting the positions indicated with the integer. As already introduced, we use Φ[I] for the sum of the probabilities of the IBD forests in $\mathcal{P}_{I}$.

If $$P_1\in \mathcal{P}_{I}$$ and $$P_2\in \mathcal{P}_{I'}$$ are separate IBD forests, implying that *I* and $I^{\prime }$ have disjoint supports, we write $$P_1\cdot P_2$$ for their combination (i.e., intersection as sets). Using the definition in Sect. 3 of the dot product of indexings, we see that $$P_1\cdot P_2 \in \mathcal{P}_{I\cdot I'}$$. Furthermore, if $$P_1$$ and $$P_2$$ are not necessarily separate, while *I* and $I^{\prime }$ still have disjoint supports, we define the *wedge product* $$P_1\wedge P_2$$ as follows: It is an IBD forest consisting firstly of all the IBD trees of $$P_1$$. Secondly, for all IBD trees in $$P_2$$ and all paths in those IBD trees, it includes all edges to the focus node from where the path either starts at its founding node or contains a node in $$P_1$$. In the second case, the path is “hijacked" to the corresponding path in $$P_1$$, i.e., it follows the path in $$P_1$$ backwards to a founding node. Note that the paths in $$P_1$$ and $$P_2$$ might have different choices of parent nodes at the hijacking node, and it is the choice of $$P_1$$ that prevails. In Fig. 2, if $$P_1\in \mathcal{P}_{00xx}$$ is the red IBD tree and $$P_2\in \mathcal{P}_{xx00}$$ is the green IBD tree, then $$P_1\wedge P_2\in \mathcal{P}_{0010}$$ is the IBD forest in panel D.

Note that while the wedge product of indexings defined in Sect. 3 is a set of indexings, the wedge product of IBD forests is a single IBD forest. Considering the possibilities and the definitions, we get the following Lemma:

### Lemma 2

If $$P_1\in \mathcal{P}_{I}$$, $$P_2\in \mathcal{P}_{I'}$$, and *I* and $I^{\prime }$ have disjoint supports, we have that

$$ P_1\wedge P_2 \in \bigcup _{J\in I\wedge I'}\mathcal{P}_{J}. $$

Now, assume *I* and $I^{\prime }$ are indexings with disjoint supports. Assume $$P\in \mathcal{P}_{J}$$ for some $$J\in I\wedge I'$$, and let $P^{\prime }$ be the restriction of this IBD forest to the paths ending in the focus nodes in the support of *I*. Then $$P'\in \mathcal{P}_{I}$$. Any inheritance pattern r∈P must necessarily also respect $P^{\prime }$, so $r\in P^{\prime }$, and $P\subseteq P^{\prime }$. We get that

28 $$\begin{aligned} \Phi [I\wedge I'] = \sum _{J\in I\wedge I'}\Phi [J] = \sum _{J\in I\wedge I'}\sum _{P\in \mathcal{P}_{J}}\Pr (P) \le \sum _{P'\in \mathcal{P}_{I}}\Pr (P') = \Phi [I]. \end{aligned}$$

Equation (28) can be viewed as a weaker form of the product theorem, Theorem 1, for which we now provide the proof:

### Proof

We can write

29 $$\begin{aligned} \Phi [I]\Phi [I']= &  \left( \sum _{P_1\in \mathcal{P}_{I}}\Pr \left( P_1\right) \right) \left( \sum _{P_2\in \mathcal{P}_{I'}}\Pr \left( P_2\right) \right) = \sum _{(P_1, P_2) \in \mathcal{P}_{I}\times \mathcal{P}_{I'}}\Pr \left( P_1\right) \Pr \left( P_2\right) \nonumber \\= &  \sum _{J\in I\wedge I'}\left( \sum _{ \begin{array}{c} (P_1, P_2) \in \mathcal{P}_{I}\times \mathcal{P}_{I'}\\ P_1\wedge P_2 \in \mathcal{P}_{J} \end{array} } \Pr \left( P_1\right) \Pr \left( P_2\right) \right) \end{aligned}$$

where we use Lemma 2 in the last equality. If $J=I\cdot I^{\prime }$, we get (see below)

$$\begin{aligned} &  \sum _{ \begin{array}{c} (P_1, P_2) \in \mathcal{P}_{I}\times \mathcal{P}_{I'}\\ P_1\wedge P_2 \in \mathcal{P}_{I\cdot I'} \end{array} } \Pr \left( P_1\right) \Pr \left( P_2\right) = \sum _{ \begin{array}{c} (P_1, P_2) \in \mathcal{P}_{I}\times \mathcal{P}_{I'}\\ P_1\wedge P_2 \in \mathcal{P}_{I\cdot I'} \end{array} } \Pr \left( P_1\wedge P_2\right) \\ &  \qquad \qquad = \sum _{P_3\in \mathcal{P}_{I\cdot I'}}\Pr (P_3) = \Phi [I\cdot I'] \end{aligned}$$

The reason for the first equality above is that, when $P_1\wedge P_2\in \mathcal{P}_{I\cdot I'}$, the IBD forests $P_1$ and $P_2$ are separate. We have $P_1\wedge P_2 = P_1\cap P_2$, and the number of edges in $P_1\wedge P_2$ is the sum of the number of edges in $P_1$ and $P_2$. The second equality follows because $P_1\wedge P_2=P_3$ establishes a one-to-one correspondence between the two sets summed over.

For the remaining terms of the sum of Eq. (29) we will establish that

30 $$\begin{aligned} \sum _{ \begin{array}{c} (P_1, P_2) \in \mathcal{P}_{I}\times \mathcal{P}_{I'}\\ P_1\wedge P_2 \in \mathcal{P}_{J} \end{array} } \Pr \left( P_1\right) \Pr \left( P_2\right) \le \Phi [J] \end{aligned}$$

As the sum in Eq. (30) is clearly non-negative, proving Eq. (30) will prove the theorem.

Consider the terms in the sum of Eq. (30) where $P_1$ has some fixed value. The computation $P_1\wedge P_2 = P_3$ then establishes a map from $\mathcal{P}_{I'}$ to $\mathcal{P}_{J}$. Fix a $P_3$ in the image of this map, and consider the set $\mathcal{S}$ of all $P_2$ such that $P_1\wedge P_2=P_3$. We then have

31 $$\begin{aligned} \sum _{P_2\in \mathcal{S}}\Pr (P_1)\Pr (P_2) \le \Pr (P_3). \end{aligned}$$

The reason is the following: Assume there are $k_1$ edges in $P_1$, and an additional $k_2$ edges in $P_3$. Then all the elements of $\mathcal{S}$ need to respect the extra $k_2$ segments. This means that

$$ \sum _{P_2\in \mathcal{S}}\Pr (P_2)\le 2^{-k_2} $$

which proves Eq. (31). We can now sum Eq. (31) over all $P_1\in \mathcal{P}_{I}$ to get that the left-hand side of Eq. (30) is bounded above by the sum of $\Pr (P_3)$ for some subset of the $P_3\in \mathcal{P}_{J}$. This implies Eq. (30). □

## Discussion and conclusion

In this paper we revisit a classical question in genetic relatedness: Given a set of identity coefficients, can it arise from an actual pedigree? Previous results have largely been confined to special cases, like a pair of noninbred individuals, and to the best of our knowledge, no general results have previously been found.

We have presented a new framework for exploring the space of identity coefficients, based on a compact *indexing* notation for certain generalized IBD patterns. Using the Product Theorem (Theorem 1) for such indexings we identified three unattainable regions in the pairwise case, each defined by a quadratic inequality in the nine coefficients $(\Delta _1, \dotsc , \Delta _9)$. It should be emphasized that we do not believe this to be a complete characterization of the space. Empirical evidence from simulations (not shown) suggests that additional large unattainable regions remain to be described. In particular we conjecture that Thompson’s inequality $\Delta _8^2 \ge 4\Delta _7\Delta _9$ holds for any pairwise relationship, not only between noninbred individuals.

Complementary to the question studied here is the construction problem: Given a point that does not lie in any unattainable region, how to construct a pedigree that produces it? Again, this has been satisfactory solved only in the noninbred case, where explicit constructions show that every point outside the unattainable region, is indeed realizable in a (possibly infinite) pedigree (Karigl 1984; Vigeland 2020). We are not aware of any similar procedures for realizing general sets of coefficients $(\Delta _1,\dotsc ,\Delta _9)$.

Another interesting question raised by our findings concerns the *estimation* of identity coefficients from data (Graffelman et al. 2025; Laporte et al. 2017; Csűrös 2014; Korneliussen and Moltke 2015; Vigeland 2021). In the noninbred case, maximum likelihood estimation of $(\Delta _7,\Delta _8,\Delta _9)$ may be constrained to the attainable part of the triangle, i.e., where $\Delta _8^2 \ge 4\Delta _7\Delta _9$. By analogy, when estimating the full vector $(\Delta _1,\dotsc ,\Delta _9)$ one may impose constraints that exclude all known unattainable regions. Note that the constraints do not apply when estimating *realized identity coefficients*, i.e., the actual proportions of the genome that is IBD in various ways in a particular case.

On a final note, our proof of the product theorem applies to all allelic pedigrees, not only those arising from ordinary pedigrees. This suggests that there may exist restrictions in the identity coefficients from ordinary pedigrees that cannot be proven within our framework.
